# Phenotypic Heterogeneity in Dementia: A Challenge for Epidemiology and Biomarker Studies

**DOI:** 10.3389/fpubh.2018.00181

**Published:** 2018-06-19

**Authors:** Joanne Ryan, Peter Fransquet, Jo Wrigglesworth, Paul Lacaze

**Affiliations:** Department of Epidemiology and Preventive Medicine, Monash University, Melbourne, VIC, Australia

**Keywords:** Alzheimer's disease, biomarkers, clinical symptoms, diagnosis, dementia, heterogeneity, pathophysiology

## Abstract

Dementia can result from a number of distinct diseases with differing etiology and pathophysiology. Even within the same disease, there is considerable phenotypic heterogeneity with varying symptoms and disease trajectories. Dementia diagnosis is thus very complex, time-consuming, and expensive and can only be made definitively post-mortem with histopathological confirmation. These inherent difficulties combined with the overlap of some symptoms and even neuropathological features, present a challenging problem for research in the field. This has likely hampered progress in epidemiological studies of risk factors and preventative interventions, as well as genetic and biomarker research. Resource limitations in large epidemiologically studies mean that limited diagnostic criteria are often used, which can result in phenotypically heterogeneous disease states being grouped together, potentially resulting in misclassification bias. When biomarkers are identified for etiologically heterogeneous diseases, they will have low specificity for any utility in clinical practice, even if their sensitivity is high. We highlight several challenges in in the field which must be addressed for the success of future genetic and biomarker studies, and may be key to the development of the most effective treatments. As a step toward achieving this goal, defining the dementia as a biological construct based on the presence of specific pathological features, rather than clinical symptoms, will enable more precise predictive models. It has the potential to lead to the discovery of novel genetic variants, as well as the identification of individuals at heightened risk of the disease, even prior to the appearance of clinical symptoms.

## Introduction

Dementia is a major public health problem, with enormous social and economic costs, and substantial burden for the individual, their caregiver and families (1). By 2050, it is estimated that over 130 million people will be living with dementia (2). This sharp increase from the 2015 estimates of 48 million reflects not only the aging population worldwide, but the current lack of effective treatments or cures. The results of drug trials to slow or halt the progression of dementia have so far been unsuccessful (3). This emphasizes the need for more research into the etiology of the diseases which cause dementia, with better characterization of genetic and environmental risk factors (4). There is also an increasing push to identify valid disease biomarkers, which would aid in diagnosis, and could be used to predict individuals at future risk (5).

## Challenges with diagnosis

Dementia is an overarching term used to describe a group of symptoms that results in severe long-term decline in cognitive function that is significant enough to affect daily function (6). Dementia can result from a number of complex disorders which damage the brain. The most common includes Alzheimer's disease (AD), vascular dementia, frontotemporal dementia, dementia with Lewy bodies, and Parkinson's disease. Typical symptoms of dementia can include a decline in memory, language deficits, and impaired visuospatial skills, as well as a loss of executive function and attention. Associated mood and behavioral disturbances, including delusions, are also frequent (6). However the exact symptoms a person experiences depends on the disease that is causing dementia, as they are distinct diseases with differing etiology and pathophysiology. Symptoms also depend on the parts of the brain that are damaged and the complexity of these conditions is such that even within common underlying conditions, presentation of symptoms differs between individuals (7). For example, there are now classifications of both typical and atypical AD (8). Further, these diseases exist on a continuum of severity and with varying disease trajectories (9). When mild, dementia can be dismissed as “normal” age-related cognitive decline, and some individuals are able to mask symptoms in the early stages (7). The extent to which dementia progresses is also highly variable. Further, given the common behavioral and mood disturbances, dementia can also be misdiagnosed as symptoms of a psychiatric disorder (10). This presents an important challenge for the field (7).

In the absence of clear biomarkers, dementia diagnosis is very challenging. Neuropsychological evaluation with profiles of cognitive strengths and weaknesses are used by both clinicians and researchers to define the likely form of dementia. This information is used in combination with reports of clinical symptoms, the results of blood tests and neuroimaging, and is in accordance with diagnostic criteria which are continually evolving (11). As such, diagnosis is often a very expensive, long and time-consuming process which does not always result in a clear outcome. The heterogeneity in symptoms within different diseases, combined with the overlapping features (both symptoms and neuropathology) across many of the diseases (Table 1) further complicates the issue. However the importance of early and accurate differential diagnosis of the underlying dementia condition is crucial. It has implications for prognosis, longer term health planning, and heritability, as well as symptom management, which could potentially be made worse by the use of incorrect treatment (20, 21). Given the continual advances in disease-modifying treatments, it also will have implications for future therapeutics (22).

**Table 1:** Common neurodegenerative disorders characterized by dementia symptoms in older individuals, and characteristic features.

**Condition**	**Estimated frequency of dementia cases^a^**	**Clinical symptoms**	**Neuropathology**	**Genetics**
Alzheimer's disease (11)	Most frequent, 60–70%.	Memory problems and other cognitive domains can also be affected (e.g., problem solving, finding words, making decisions)	β-amyloid protein plaques & neurofibrillary tangles composed of tau protein. Brain atrophy	Amyloid precursor protein (APP), Presenilin-1 &-2 (*PSEN1, PSEN2*), Apolipoprotein E (ApoE)
Vascular dementia (12)	10–20%. Multiple subtypes (e.g. multi-infarct dementia, subcortical vascular dementia)	Impaired judgement or decision making. Varies depending on position of strokes/infarcts	Blood vessel & vascular related brain damage. Caused by chronic reduced blood flow to the brain, usually as a result of a stroke or a series of strokes	Very rare: cerebral autosomal dominant arteriopathy with subcortical infarcts & leukoencephalopathy (*CADASIL*)
Frontotemporal dementia (13)	10%. Multiple subtypes (e.g., behavioral-variant frontotemporal dementia & primary progressive aphasias)	Changes in personality & behavior, & difficulties with speech. Behavioral variant: progressive deterioration of personality, social comportment & cognition. Primary progressive aphasia: impairments in language production & speech, impaired comprehension	Atrophy in one or both of the frontal or temporal lobes. Highly heterogeneous depending on subtype. Can include Pick bodies, which are positive for Tau and ubiquitin proteins.	Progranulin (*GRN*), Microtubule-associated protein tau (*MAPT*), Chromosome 9 open reading frame 72 (*C9orf72*), Valosin-containing protein (*VCP*) (14)
Dementia with Lewy bodies (15)	5% (16)	Confusion, attentional deficits in visuospatial function. Apathy & hallucinations are common. Absence of motor alterations seen in Parkinson's disease	Abnormal aggregates of α-synuclein proteins, which form spherical structures (Lewy bodies) in nerve cells. β-amyloid and tau accumulation	Rare autosomal dominant inheritance: Alpha synuclein (*SNCA*), leucine-rich repeat kinase family (*LRRK2*), glucocerebrosidase (*GBA*)
Parkinson's disease (17)	Up to 80% of patients with Parkinson's disease progress to dementia	Motor alterations including tremor, rigidity, bradykinesia, changes in gait. As it progresses, dementia like that seen in dementia with Lewy bodies or AD is common	Accumulation of α-synuclein aggregates in diverse brain regions, often begins in the substantia nigra. Result in degeneration of dopaminergic neurons. β-amyloid and tau accumulation	Rare autosomal dominant inheritance: *SNCA* and *LRRK2* genes

a *Kosunen et al. (18); Brayne et al. (19)*.

## Alzheimer's disease

The most common form of dementia is AD, an insidious and incapacitating neurodegenerative disorder which accounts for ~60% of all dementia cases (23). The defining pathological features of AD are the presence of two proteins in the brain, amyloid, and tau. Accumulated amyloid beta (β) peptides clump together forming extracellular neurotic plaques, while hyper phosphorylated TAU proteins form intracellular neurofibrillary tangles (24). A definitive diagnosis of AD thus requires histopathologic confirmation via post-mortem. In living individuals, AD is diagnosed as probable or possible according to set criteria (often DSM) by a panel of expert clinicians who review a range of documentation (25). The guidelines established by the National Institute of Neurological and Communicative Disorders and Stroke and the Alzheimer's Disease and Related Disorders Association work group (NINCDS-ADRDA), updated in 2011, are the most frequently used for dementia diagnosis (11). An expert panel review the results of extensive neuropsychological testing, detailed medical history, blood tests and imaging, such as magnetic resonance imaging (MRI), positron emission tomography (PET), and/or computerized tomography (CT) and reach a consensus.

## Overlapping features and misdiagnosis

However studies have shown that a significant proportion of individuals diagnosed with probable/possible AD by experts, do not display the hallmark neuropathological criteria for AD on post-mortem examination (26). In many other cases, more than one form of dementia is identified (“mixed dementia”), and this becomes increasingly more common in later life (27). Vascular dementia is caused by stroke and/or small vessel disease and includes a number of different sub-types (12). It occurs frequently with AD and the presence of both could exacerbate the development of dementia compared with either condition alone (28). Coexistent Parkinson's disease changes also occur relatively frequently in individuals with AD (29). Dementia with Lewy bodies sometimes co-occurs with AD or vascular dementia or can be misdiagnosed as these conditions depending on the presence of symptoms of cognitive impairment or Parkinsonism (30). In fact, Dementia with Lewy bodies and Parkinson's disease are now considered as a continuum of the same disease (Lewy body dementias), with Dementia with Lewy bodies being an early manifestation in patients with Parkinson's (15).

Adding further to these complexities is the overlap in neuroanatomical features of these disorders (Table 1). The hallmark features of AD are the accumulation of amyloid-β and tau protein, yet neither is sufficient to cause dementia nor unique to this disease (31). Tau may be present from early adulthood and could only become problematic once amyloid accumulates (32). Even then, around 30% of people may have amyloid accumulation without any obvious clinical symptoms (33, 34). Dementia with Lewy bodies also shares the neuropathology characteristics of amyloid-β and tau (REF), and the latter is also found in other neurodegenerative conditions such as chronic traumatic encephalopathy (30). Parkinson's disease and frontotemporal dementia both involve tau alterations, but these are a loss of function rather than phosphorylation (35). Likewise, hallmark characteristics of Lewy body dementias, such as α-synuclein inclusions, are also found in many cases of AD (36).

Increasingly evidence from studies investigating neuropathology and molecular genetics has demonstrated that clinical symptoms (phenotype) are not always tightly linked with etiology, as they can be influenced by a variety of other factors including prior experience, cognitive reserve, and epigenetics (37). Studies of several autosomal dominant dementias indicate that the presenting clinical phenotype may vary widely, even for those individuals with the same causative mutation. For example, mutations in the *PSEN1* gene are considered almost deterministic for earlier onset AD, yet there is considerable heterogeneity in the clinical expression of neurological features (38). This can include behavioral and psychiatric symptoms which can sometimes reflect frontotemporal dementia or dementia with Lewy bodies (38). Another example is a very rare autosomal dominant neurodegenerative disorder, frontotemporal dementia and Parkinsonism linked to chromosome 17 (*FTDP-17*) which has different phenotypes, even within families carrying the exact same mutation (39). The most established genetic risk factor for late-onset AD is the *APOE* ε4 allele, and this is also over-represented in sporadic Lewy body dementias compared with controls (40).

Other people have argued that the different dementia conditions are highly related conditions with a continuous range of abnormalities (41), although genetically and epigenetically they are distinct. Indeed, dementia with Lewy bodies has been shown to be similar genetically to AD, while AD and Parkinson's disease were only very weakly correlated (42). Similar aberrant changes in DNA methylation patterns have also been found in individuals with different forms of dementia (41). However the vast majority of genetic and epigenetic patterns are unique to each disease (37). Further, given the potential inaccuracies in diagnosing dementia, overlapping patterns may also reflect, at least in part, inaccuracies in how the conditions have been defined (discussed further below).

The inherent difficulties in diagnosing dementia, as well as the overlapping symptoms and even neuropathological features, presents a complex and challenging problem for research in the field. This is likely to have hampered progress in genetic and biomarker studies to date, as well as epidemiological studies of risk factors and preventative interventions.

## Problems with inaccurate phenotyping

Genetic and biomarker studies rely on accurate phenotypes and diagnosis (43). Most genetic risk variants identified from such studies are either rare with moderate effect sizes or common with very small effect sizes (44). Large samples are thus needed to have sufficient power to detect true associations, especially at genome-wide significance levels (45). Mixing together diseases with different etiology, pathophysiology and potentially different genetic architecture, is obviously problematic for the investigation of novel genetic variants, diluting out any signals (43). As an example, new genetic loci identified as being associated with clinically-defined AD, were not found to be associated with AD neuropathology at postmortem (46). Similar problems are likely to be plaguing new biomarker discovery. When biomarkers are identified, if they are in fact reflective of etiological heterogeneous disease states, they will have low specificity for any utility in clinical practice, even if their sensitivity is high (47). These issues are exacerbated by the challenges in selecting unaffected controls who are without dementia. AD for example has a very long pre-symptomatic phase (48), meaning that individuals without dementia in the “control” group, may be free of clinical symptoms, but could already have the disease. Together these issues may help explain the lack of substantial progress in this field to date.

## Unique challenges for large cohorts

Epidemiological cohort studies of dementia, often with the aim of identifying risk and protective factors for the disease (49, 50), are confronted with many of these challenges. Risk factors identified as being associated with cognitive decline and AD diagnosed solely on the basis of clinical symptoms, may in fact not be associated with AD pathology (46). Diagnosing dementia is expensive and time-consuming, which is compounded when undertaken on a larger scale. As a result, studies often only collect relatively sparse phenotypic data, without imaging, blood measures or other biological markers (51).

In recent years there have been widely commended efforts to increase uniformity around the diagnostic criteria for dementia and the underlying construct. The vast majority of publications in good quality journals now define probable AD using clinical criteria by the National Institute on Aging-Alzheimer's Association (NIA-AA) (11). However this criteria predominantly lists recommendations rather than requirements, with the acknowledgment that not all clinicians will have access to the results of the full range of tests, which are time consuming and expensive to obtain. This criteria also includes evidence of neurodegeneration, and thus recommends where possible, that MRI is used to assess cerebral atrophy, but there are no strong criteria regarding other neuropathological changes. With published studies, there is rarely detailed information concerning the information that was obtained to support a dementia diagnosis, and thus difficult for the reader to assess the strength of evidence for these diagnoses. Many studies instead broadly define dementia, and determine risk factors for this heterogeneous condition, which has obvious limitations (as discussed above).

A large number of other studies use less reliable measures of dementia, such as self-reports, linkage data (52), or community diagnoses, with no additional clinical evidence sought to confirm and establish dementia diagnosis (51, 53). This has obvious problems and would increase both the false positives and false negatives. ICD coding is also still frequently used, but has well-documented limitations (54). In other cases, exact diagnostic criteria is not stated (41). Together such studies are likely to be plagued by misclassification bias which would make it more difficult to identify true associations.

## Future directions

Currently the methods for identifying and delineating different dementia sub-types are imperfect and not scalable. For research to advance in this area there is a need for better definitions, with clearly established guidelines for the minimal information which must be collected data, and diagnostic markers are required to improve classification of the underlying form of dementia and at a level which is standardized and scalable for large studies.

Deep phenotyping is considered to be the key to advancing genetic studies (55), and this is not just unique to dementia, although it may be one of the most challenging areas. Descriptions of disease phenotypes often do not capture the full diversity of clinical and even pathophysiological manifestations. Advancing research in this area may require sub-categorization of the disease into more homogenous groups or disease states, which would permit increased precision (18, 46). Indeed, very recently there have been calls from the NIA-AA working group to establish a new research framework where AD is defined as a pathophysiological construct, rather than a clinical syndrome (56). While AD is often described by its clinical symptoms, it was identified and initially defined by its neuropathological features, namely the build-up in the brain of β-amyloid (Aβ) protein plaques and neurofibrillary tangles composed of aggregates of hyperphosphorylated TAU protein (11). The presence of these protein enables a definitive diagnosis of AD to be made post-mortem and there are now validated *in vivo* biomarkers for these. Using PET combined with MRI (to assess brain atrophy), the accumulation of amyloid-β and phosphorylated tau can be ascertained (57, 58). Defining AD as a biological construct based on the presence of these imaging biomarkers, will enable the generation of more precise predictive models for this specific neuropathological processes. This will shift away from the focus on clinical symptoms of the disease which are phenotypically heterogeneous, as discussed above, and thus problematic for biomarker and epidemiological studies.

## Conclusion

The results of drug trials to slow or halt the progression of dementia have so far been unsuccessful (3), raising at least two important issues. Current treatments and interventions are unlikely to be effective in individuals with overt disease symptoms. However they could be effective if targeted very early in the disease process, before the appearance of clinical signs. Hence the need for clear biomarkers which would permit timely diagnosis and accurate characterization of the underlying condition resulting in dementia. Secondly, disease prevention is recognized as increasingly important, given the current lack of therapeutics. This is particularly pertinent for individuals identified at high-risk of the disease. This stresses the need for accurate risk prediction models, and thus the identification of the full range of genetic risk variants, as well as environmental factors through large epidemiological studies. This will also facilitate the categorization of subgroups within the population most suited for studies of new pharmacological and non-pharmacologic interventions. Adding to this is the increasing focus on precision medicine more generally.

Accurately determining the condition resulting in dementia is critical for research, including epidemiological, genetic, and biomarker studies (46). It is also of particular importance for treatment and prevention trials. Currently there are many challenges with diagnosing dementia, and as such it is a long, complicated and costly task, and misdiagnosis remains an issue. The emergence of new disease biomarkers will have a considerable impact on clinical diagnostic procedures. However, advances in biomarker research have been limited the inability to define a “clear” homogenous dementia phenotype with current biomarkers having considerable overlap with a number of dementia conditions. This creates a circularity problem which is difficult to resolve. However these challenges must be addressed if the likelihood of success for future genetic and biomarker studies is to increase. As an initial step, the focus on neuropathological markers of dementia and defining dementia as a biological construct will enable more accurate characterization of risk factors specific for this disease, shifting the definition from syndromal to biological (56, 59). It has the potential to lead to the discovery of novel genetic variants, the identification of readily accessible peripheral biomarkers reflective of these neuropathological processes, as well as the identification of individuals at heightened risk of the disease, even prior to the appearance of clinical symptoms (60). This will also become an increasingly important issue as new drug treatments are developed (61).

## Author contributions

JR and PL conceived the idea. JR wrote the first draft of the manuscript. All authors contributed to revising the final manuscript.

### Conflict of interest statement

The authors declare that the research was conducted in the absence of any commercial or financial relationships that could be construed as a potential conflict of interest.
